# Evaluating Phenotypic and Transcriptomic Responses Induced by Low-Level VOCs in Zebrafish: Benzene as an Example

**DOI:** 10.3390/toxics10070351

**Published:** 2022-06-27

**Authors:** Chia-Chen Wu, Jessica R. Blount, Alex Haimbaugh, Samantha Heldman, Jeremiah N. Shields, Tracie R. Baker

**Affiliations:** 1 Department of Global and Environmental Health, University of Florida, Gainesville, FL 32610, USA; chiachenwu@ufl.edu; 2 Institute of Environmental Health Sciences, Wayne State University, Detroit, MI 48202, USA; jrblount@med.wayne.edu (J.R.B.); alexhaim@wayne.edu (A.H.); smheldman@wayne.edu (S.H.); jshields@wayne.edu (J.N.S.); 3 Department of Pharmacology, School of Medicine, Wayne State University, Detroit, MI 48202, USA

**Keywords:** volatile organic compounds, benzene, zebrafish, transcriptomics

## Abstract

Urban environments are plagued by complex mixtures of anthropogenic volatile organic compounds (VOCs), such as mixtures of benzene, toluene, ethylene, and xylene (BTEX). Sources of BTEX that drive human exposure include vehicle exhaust, industrial emissions, off-gassing of building material, as well as oil spillage and leakage. Among the BTEX mixture, benzene is the most volatile compound and has been linked to numerous adverse health outcomes. However, few studies have focused on the effects of low-level benzene on exposure during early development, which is a susceptible window when hematological, immune, metabolic, and detoxification systems are immature. In this study, we used zebrafish to conduct a VOC exposure model and evaluated phenotypic and transcriptomic responses following 0.1 and 1 ppm benzene exposure during the first five days of embryogenesis (n = 740 per treatment). The benzene body burden was 2 mg/kg in 1 ppm-exposed larval zebrafish pools and under the detection limit in 0.1 ppm-exposed fish. No observable phenotypic changes were found in both larvae except for significant skeletal deformities in 0.1 ppm-exposed fish (*p* = 0.01) compared with unexposed fish. Based on transcriptomic responses, 1 ppm benzene dysregulated genes that were implicated with the development of hematological system, and the regulation of oxidative stress response, fatty acid metabolism, immune system, and inflammatory response, including *apob*, *nfkbiaa*, *serpinf1*, *foxa1*, *cyp2k6,* and *cyp2n13* from the cytochrome P450 gene family. Key genes including *pik3c2b*, *pltp*, and *chia.2* were differentially expressed in both 1 and 0.1 ppm exposures. However, fewer transcriptomic changes were induced by 0.1 ppm compared with 1 ppm. Future studies are needed to determine if these transcriptomic responses during embryogenesis have long-term consequences at levels equal to or lower than 1 ppm.

## 1. Introduction

Benzene, toluene, ethylbenzene, and xylene (BTEX) is a common volatile organic compound (VOC) mixture associated with the petrochemical industry. They are common constituents of crude oil and thus are important raw materials or intermediates in the manufacture of many chemicals. They are also byproducts of incomplete combustion and can be released into the air during oil and gas operations. Despite legislation and enforcement, anthropogenic sources such as vehicle exhaust, gas emissions from manufacturing and chemical facilities, and spills or leaks involving gasoline and diesel fuel still occur and can lead to ambient levels ranging from 0.1 to 100 µg/m^3^ in industrialized urban regions [1,2,3,4]. Environmental BTEX contribute significantly to poor air quality and pose a public health hazard across the globe [5,6].

Among the BTEX mixture compounds, benzene is the most volatile compound and has been linked to numerous adverse health outcomes that involve neurological, respiratory, cardiovascular, endocrine, renal, hepatic, immunological, and hematological systems [7]. Developmental exposure to benzene, particularly, needs more attention since it has been associated with abnormal birth outcomes in humans at non-occupational settings [8,9]. Emerging epidemiological studies indicate that children exposed to environmental benzene have elevated abnormalities in blood cells formation and hematologic functions compared with unexposed children [10,11,12]. The mechanisms of benzene-induced toxicity are not completely understood, but are believed to be associated with the metabolites of benzene, such as benzene oxide, phenol, hydroquinone, and catechol [13]. These metabolites cause damage in hematopoietic cells, lead to chromosomal aberration, produce oxidative stress, damage the immune system, and alter gene expression and epigenetic regulation [14,15,16]. Children and fetuses are more susceptible to low-level benzene exposure than adults due to ongoing development of host defenses, detoxification mechanisms, and hematopoietic and metabolic systems [17].

The lack of information on effects of developmental exposure to low-level benzene necessitates the use of a high-throughput animal model to address exposure concerns for this pervasive chemical. Due to the short generation times, high fecundity, and ease of husbandry, zebrafish is an excellent model for developmental toxicity screening for VOC. Since zebrafish share approximately 70% homologous genes with humans, results from toxicology assays using this vertebrate model are translatable to human health and disease outcomes [18]. In addition, zebrafish hematological and immune systems are similar to mammals [19,20], and the molecular pathways governing developmental hematopoiesis are highly conserved between zebrafish and mammals [21]. The innate immune system matures during zebrafish embryonic development and the full adaptive immunity is functional after 4–6 weeks postfertilization. Larval zebrafish life stages are suitable for studying the development of innate immunity and the regulation of the inflammatory response [22].

In this study, we evaluate low levels of benzene exposure (0.1 and 1 ppm) during the first five days of embryogenesis to identify the potential-induced phenotypic and transcriptomic changes and their associations with diseases and pathways implicated in human benzene exposure. To maintain consistent and rigorous control over the benzene concentrations during the exposure period, chemical exposures were conducted in sealed containers with daily water changes including renewal of the exposure chemicals. The benzene levels in water and whole larvae body were monitored. The results of this study will provide molecular signatures of benzene exposure in zebrafish that will guide and add dimension to the interpretation of VOC exposure studies in mammalian and human tissues and populations.

## 2. Methods

### 2.1. Fish Husbandry

The zebrafish used in this study were wild-type AB strains kept in 27–30 °C reverse osmosis water buffered with 60 mg/L Instant Ocean Salts in a recirculating system (Aquaneering Inc., San Diego, CA, USA) on a 14 h:10 h light/dark cycle. Twice daily, adult fish were fed Aquatox Fish Diet (Zeigler, PA, USA) and spirulina flakes (Zeigler, PA, USA). To obtain embryos for VOC exposure experiment, adult fish were housed in spawning tanks with a sex ratio of two females: one male. Four hours post fertilization (hpf), embryos were collected, cleaned with 58 ppm bleach (diluted from Chlorox bleach, the Clorox Co., Oakland, CA, USA) for 5 min, and rinsed with egg water that was made up of 600 mg/L Instant Ocean salts (Spectrum Brands, Madison, WI, USA) in RO water. The clean eggs were used for the exposure experiment. The Institutional Animal Care and Use Committee at Wayne State University approved these zebrafish protocols on 14 October 2020 (protocol #IACUC-19-02-0938) in accordance with the Guide for the Care and Use of Laboratory Animals [23].

### 2.2. Chemical Exposure

#### 2.2.1. The Stock Chemicals and Dilutions

The 99.8% benzene stock (Sigma Aldrich, St. Louis, MO, USA) was serially diluted in a septum-sealed amber vial with 40 mL egg water to create 998 ppm and 99.8 ppm benzene stock dilutions. To minimize benzene loss through volatilization, benzene stock dilutions were mixed fresh daily, and caps were never removed from septum vials during or after benzene injection into egg water.

#### 2.2.2. Exposures

Embryonic benzene exposures were conducted in 30 mL glass vials with septum caps. Each vial contained 20 embryos in 20 mL of egg water per exposure level until 5 days post fertilization (dpf). To inject benzene stock dilution into the glass vials, 20 μL of egg water, the amount equal to the stock dilution about to be added, was taken out to avoid pressure imbalance. Then, 20 μL, 998 ppm, and 99.8 ppm benzene stock dilution, or egg water, was injected through the sealed septum cap directly into the egg water with a gas tight syringe to reach 1, 0.1, and 0 ppm concentrations. Vials were gently inverted several times to mix and then incubated at 28 °C.

Over the course of five days, daily deaths and hatch rates were recorded. Dead embryos were removed from the vial. Water change was conducted in each vial daily by removing 20 mL exposure solution, replenishing 20 mL fresh egg water, and injecting benzene stock solutions or egg water as on day 0. To end chemical exposure on 5 dpf, larval fish were collected, rinsed three times with egg water, and divided into 24-well plates for subsequent behavioral analysis, abnormality, and mortality screening. The exposure experiment was repeated three times with the same replicate number of embryos per concentration. Embryos of each exposure experiment were obtained from 13 to 18 spawns of different parental stocks in our fish husbandry. A total of 740 embryos were used per treatment.

### 2.3. Measurements of Body Burden

To measure the benzene uptake, 2 mL water samples, 50 embryos, and approximately 50 larval fish were collected and processed to measure benzene concentration on 1 and 5 dpf. The water samples from all three exposure experiments were collected before water change. The embryos and larval fish were sampled from two exposure experiments. Embryos or fish were placed into glass vials and euthanized by cooling the vials on ice for 30 min. Egg water was then removed completely, and methanol was added to limit benzene volatilization. Fish or eggs were weighed and quickly transferred into methanol to 2 mL screw-top vials. These vials were then sonicated for 30 min in a cup horn sonicator with the frequency of 2 s on and 1 s off at 90% amplitude in 4 °C water bath. The resultant samples were then sent to Ann Arbor Technical Services (Ann Arbor, MI, USA) and analyzed by purge and trap GC/MS according to the USEPA method 8260C [24].

### 2.4. Abnormality and Mortality Screening

All zebrafish embryos were screened for mortality daily throughout the 5-day exposure period. On day 5, the following morphological abnormalities were assessed for the live embryos: numbers of unhatched embryos, skeletal deformities, improperly inflated swim bladder, yolk sac edema, and heart edema. Chi-square analysis and Bonferroni pairwise adjustment were conducted to compare if the morphological abnormalities differ from the control treatment.

### 2.5. Behavioral Screening

On day 8, fish with swim bladders and without morphological abnormalities were selected for behavioral testing. For each exposure concentration, 32 larval fish were divided into 24-well plates, with each well housing a single fish and 2 mL of fish water. The plated fish were placed into a Noldus DanioVision Observation Chamber (Noldus Information Technology, Wageningen, The Netherlands) with a constant temperature of 28.5 ± 0.5 °C. In the observation chamber, larval movements were tracked over 24 min, with alternating light-dark cycles lasting 3 min each. Movement data, including distance traveled, velocity, turn angle, and angular velocity were integrated every 30 s. All behavioral assays took place between 14:00 and 22:00.

### 2.6. RNA Isolation

Larval fish were quickly euthanized with tricaine methanesulfonate solution and then pooled in groups of five in RNALater. RNA was isolated using the RNeasy Lipid Tissue Mini Kit (Qiagen, Hilden, Germany) according to manufacturer’s instructions. Briefly, the frozen larval pool was homogenized in 300 μL of Qiazol lysis reagent (Qiagen, Hilden, Germany) with a sterile pestle and motor (Kimble-Kontes, Vineland, NJ, USA) until no tissues were seen. Then 700 μL of Qiazol reagent (Qiagen, Hilden, Germany) and subsequently 200 μL of chloroform (Fisher Scientific, Waltham, MA, USA) were added to yield RNA partitions at the upper aqueous phase. The aqueous phase was collected and mixed with the same amount of ethanol for appropriate binding conditions. The solutions were applied to the RNeasy Mini spin column (Qiagen, Hilden, Germany), purified, and eluted in 30 μL of nuclease-free water following the manufacturer’s protocol. The concentrations of eluted RNA were measured using a Qubit 3.0 Fluorometer (Invitrogen, CA, USA). In total, 15–20 larvae per treatment were used for RNA isolation.

### 2.7. Transcriptomic Analysis

From isolated RNA, 3′ mRNA-seq libraries were prepared using QuantSeq 3′ mRNA-Seq Library Prep Kit FWD for Illumina (Lexogen, Vienna, Austria). Samples were normalized to 40 ng/μL (total input of 200 ng in 5 μL) and amplified at 17 cycles. The sample concentrations and qualities of each sample were determined using a Qubit^®^ 3.0 Fluorometer and Qubit^®^ dsDNA Broad Range Assay Kit (Invitrogen, Carlsbad, CA, USA). The libraries were run on an Agilent TapeStation 2200 (Agilent Technologies, Santa Clara, CA, USA) for quality control and sequenced on a NovaSeq 6000 (Illumina, CA, USA) with single-end 75 bp reads. Reads were aligned to *D. rerio* (Build danRer10) using the BlueBee Genomics Platform (BlueBee, Rijswijk, The Netherlands). Differential gene expression between the control and exposure treatment was evaluated using DEseq2 (available through GenePattern; Broad Institute, Cambridge, MA, USA). Genes with significant changes in expression, as defined by absolute log2 fold change value ≥ 0.75 and *p* value < 0.05, were uploaded into the Ingenuity Pathway Analysis software (IPA; QIAGEN Bioinformatics, Redwood City, CA, USA) for analysis using Ensembl gene ID as identifiers. The significant biological process and canonical pathways were defined as those with *p* values < 0.5, (or −log (*p*-value) ≥ 1.3) based on the right-tailed Fisher’s Exact Test. The z-score was generated through an IPA software to predict the activation (≥2) or inhibition (≤−2) of a pathway.

## 3. Results

**Figure 1:**
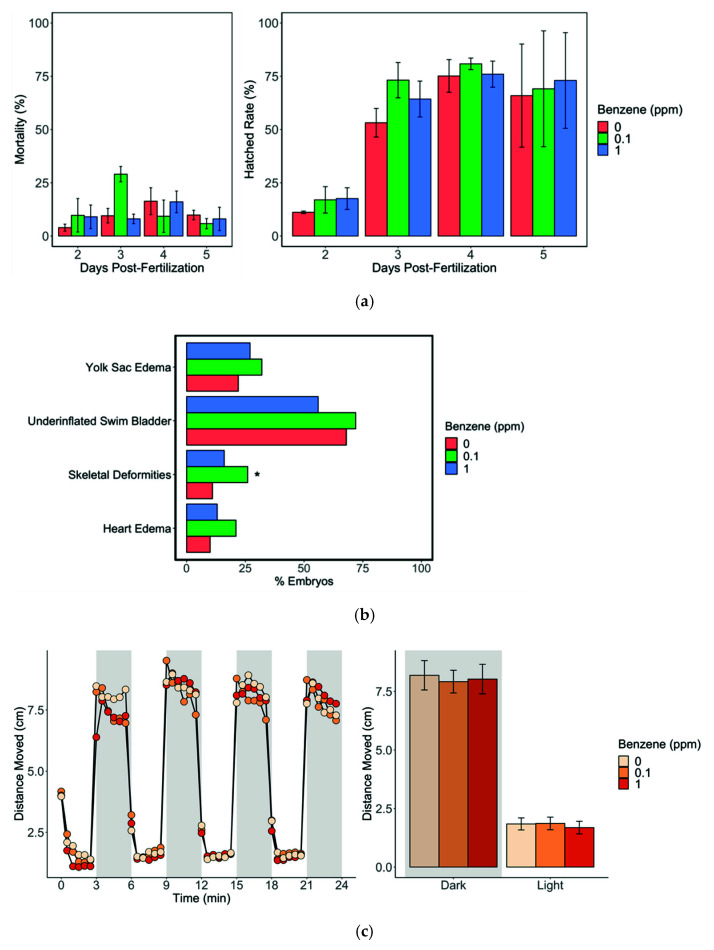
Phenotypic responses following benzene exposure from 4 h post-fertilization (hpf) to 5 days post-fertilization (dpf). (**a**) Average mortality (**left**) and hatch rate (**right**) of live embryos over the course of exposure period (n = 740 per treatment); (**b**) percentage of embryos with morphological abnormalities at 5 dpf per treatment (n = 134, 149, 146 for 0, 0.1, and 1 ppm benzene); or (**c**) average distance moved by larval zebrafish during light and dark cycles (n = 32 per treatment). * Condition is significantly different from control (0 ppm; *p* < 0.05).

We measured the benzene levels in water and larval tissues during the exposure period. At the end of day-1 exposure (before the water change), mean benzene levels in the water phase were 0.74 ± 0.3 and 0.14 ± 0.05 ppm for 1 and 0.1 ppm benzene exposures, respectively. After 5-day exposure, the mean benzene body burden was 2.0 mg/kg in the 1 ppm-exposed larval fish pool, and below the detection limit (<0.064 mg/kg) in 0.1 ppm-exposed fishes. For phenotypic responses, neither 0.1 ppm nor 1 ppm benzene exposures affected the hatch rate or mortality compared with controls (Figure 1). On day 5, surviving fish were assessed for morphological abnormalities including skeletal deformities, underinflated swim bladders, and yolk sac and heart edemas. Only the 0.1 ppm exposure group showed twice as many skeletal deformities (26%, *p* = 0.01) and heart edema (21%, *p* = 0.06) than the control (11% and 10%, respectively). No other significant morphological differences were observed between 1 ppm-exposed and unexposed fish (Figure 1). In order to investigate the potential neurological effects of benzene exposure during development, we performed behavioral assays that track larval zebrafish movement during periods of light/dark stimulus. We observed no differences between control fish and those exposed to 1 ppm or 0.1 ppm benzene (Figure 1).

**Figure 2:**
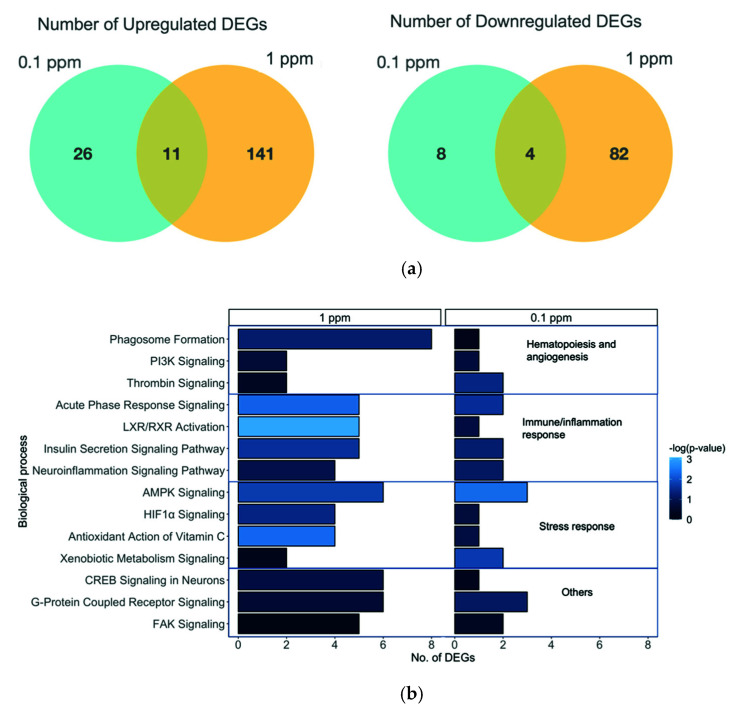
Transcriptomic responses following benzene exposure from 4 h post-fertilization to 5 days post-fertilization (dpf). (**a**) Venn diagram of the number of differentially expressed genes (DEGs) upregulated or downregulated at 0.1, 1 ppm, and both; and (**b**) number of DEGs implicated in top-rank canonical pathways.

The transcriptomic analysis uncovered 239 and 49 differentially expressed genes (DEGs) in fish exposed to 1 and 0.1 ppm benzene, respectively, with 15 transcripts overlapping between the two exposure groups (Figure 2, Table S1). All overlapping transcripts expressed in the same direction. For 1 and 0.1 ppm exposures 152 and 37 transcripts were upregulated, while 86 and 12 transcripts were downregulated, respectively. These DEGs were implicated in disorders in gastrointestinal, endocrine, reproductive, and hematological systems (Table S2). The top associated networks and canonical pathways of these dysregulated genes were involved with stress response, inflammation, and the development of the hematological and immunological system (Figure 2b). Only 1 ppm exposure was predicted to inactivate or activate some biological processes (Table S3), including decreased cognition (z = −2.4), and learning ability (z = −2.2).

Table 1 shows the DEGs of interest that were either dysregulated in both benzene concentrations or involved with multiple pathways. The DEGs shared between two benzene exposure levels included *pik3c2b*, *mep1a.2*, *chrnb2*, and *pltp*. We found the upregulation of *pik3c2b* involved most of the implicated canonical pathways. *Pik3c2b* encodes phosphatidylinositol kinases and is responsible for phosphoinositide metabolism in vertebrate tissues, which is essential for membrane trafficking and intracellular signaling cascades in numerous biological processes [25,26]. Another overexpressed gene, *mep1a.2* is orthologous to human *mep1a* and encodes alpha meprin A that has metalloendopeptidase activity. In our study, both 1 and 0.1 ppm benzene upregulated *mep1a.2* 1.7-fold higher than the control. The dysregulation of *mep1a.2* was predicted to involve intestinal inflammatory response [27] and angiogenesis effect [28]. *Chrnb2*, encoding for beta 2 nicotinic cholinergic receptor, has been implicated with impaired cognition and learning ability following 1 ppm exposure. For both benzene exposures, pathway analysis implies *chrnb2* can be involved with AMPK signaling and inflammatory response. Another shared gene that is involved with innate immune response is *pltp*, encoding phospholipid transfer protein (PLTP), which can modulate the production of proinflammatory cytokines [29]. Some shared genes were not involved with top canonical pathways but play important roles in embryogenesis, such as *chia.2* and *taf4a*. *Chia.2*, one of the genes from chitinase gene family that is essential for zebrafish development [30], was upregulated approximately two-fold higher than control following both exposures. Their encoding proteins also regulate type 2 immune responses and can be triggered during allergic rhinitis in mammals [31]. Another gene that is associated with the production of blood cells and epithelial cells in liver during embryogenesis, *taf4a*, benzene downregulated the expression compared with the control [32,33]. For 0.1 ppm exposure, *mapk11* was associated with most canonical pathways, which is involved in MAPK signaling in various cellular processes including embryogenesis and innate immunity [34].

Transcriptomic changes following 1 ppm benzene exposure were associated with detoxification processes, hematological development, and inflammation response. We detected upregulation of two genes from the cytochrome P450 (CYP) family commonly involved in the biotransformation of xenobiotics, *cyp2k6* (orthologous to human cyp2w1) and *cyp2n13* [35,36]. Many genes from the solute carrier (slc) family were also dysregulated, including downregulation of *slc2a1a* and *slc9a5*, as well as upregulation of *slc23a3*, *slc27a2b*, and *slc26a3.2.* These genes are implicated with hematological diseases or inflammatory responses. Several DEGs are implicated with hematopoietic neoplasm, such as the downregulation of *antxr;* it encodes the anthrax toxin receptor and can inhibit vascular development in zebrafish [37]. The *Apob* expression also can alter angiogenesis by mediating the expression of the receptors of the vascular endothelial growth factor [38]. Among the genes of interest that were exclusively differentially expressed following 1 ppm exposure, *c3a.3* had the highest fold change. This gene is orthologous to human complement C3 and is essential for innate immunity [39]. Along with *c3a.3*, another complement component, *c2* (*si:ch1073-280e3.1*), was also upregulated following 1 ppm exposure. Two cytokine-related genes, *il15* and *ifih1*, encoding interleukin and interferon, respectively, are implicated with innate immune response and downregulated following 1 ppm exposure. A negative regulator of immunity responses [40], *nfkbiaa*, encoding the inhibitor of nuclear factor κB (NF-κB) transcription factors, was upregulated following 1 ppm benzene exposure. Some 1 ppm upregulated genes are responsible for multiple functions, such as *foxa2* and *serpinf1*. *Foxa2* encodes for transcription factors of the Forkhead box A family and is responsible for glucose and lipid homeostasis in liver [41] and adipose cells [42]. In addition, it is also involved in the development of embryonic structures [43], maturation of intestinal goblet cells [44], and modulation of inflammation response in skeletal and muscle cells [45]. The other gene, *serpinf1*, is also involved in hepatic lipid metabolism [46], and can be mediated by interleukin and vascular endothelial growth factors to inhibit angiogenesis [47].

## 4. Discussion

This is the first study to use zebrafish as a toxicological model for benzene exposure. Our results show that embryonic exposure to benzene levels as low as 0.1 ppm exerted skeletal abnormalities, and both 0.1 ppm and 1 ppm induced transcriptomic changes. Following a five-day exposure, up to 2 mg/kg benzene was detected in 1 ppm-exposed larval zebrafish pools, which can be viewed as the adsorbed dose of benzene in entire larvae tissues after metabolism. Cells in the embryonic zebrafish liver perform xenobiotic metabolism [48], and benzene metabolism can occur in the liver and bone marrow under exposure levels ranging from nanogram to milligram levels per kilogram body weight [14,49,50,51,52].

Among phenotypic responses, the only statistically significant parameter in our study was skeletal deformities following 0.1 ppm exposure. This morphological abnormality might be related to the dysregulation of *mapk11*, which is essential for controlling the initiation of dorsalization signals in zebrafish embryogenesis [53]. Among previous studies, skeletal malformations were found across species after prenatal exposure at levels larger than 47 ppm [54]. While skeletal abnormalities were not found in 1 ppm-exposed fish, more studies are needed to investigate if low-level benzene causes non-monotonic skeletal malformation during embryogenic stage.

The number of dysregulated genes implicated in the top canonical pathways indicates a monotonic dose–response relationship in transcriptomic changes. Among all, *pik3c2b* can be regarded as a key gene since it was induced by both exposure concentrations and was implicated in the most canonical pathways, including xenobiotic metabolism signaling, HIF1a signaling, AMPK signaling, FAK signaling, PI3k/Akt signaling, glucocorticoid receptor signaling, NRF2-mediated oxidative stress response, and G-protein coupled receptor signaling. Other genes from the PIK gene family, *pik3cg*, *pik3r1*, and *pik3r2*, were also differentially expressed in workers with chorionic benzene exposures [55]. For 0.1 ppm exposure specifically, *mapk11* was one of the few DEGs that was implicated in more than one pathway, including xenobiotic metabolism signaling, AMPK signaling, antioxidant action of vitamin C, and glucocorticoid receptor signaling. For 1 ppm exposure, *hsp90aa1*, *igf1*, *slc2a1*, *nfkbia*, *cyp2k6* and *cyp2n13* were implicated in multiple pathways related to oxidative stress and detoxification process. The CYP protein family is known to oxidize benzene [56]. Common CYP genes, such as *cyp2e1* and *cyp2f1* were upregulated in workers exposed to low-level airborne benzene [57]. One of the CYP enzymes, CYP2B1, was found to be destructed by benzene metabolites, phenobarbital, and then be rescued by ascorbate (vitamin C) [56]. In our study, several genes relevant to antioxidant action of vitamin C were altered by 1 ppm exposure, such as the overexpression of *nfkbia*, *plaat1*, and *slc23a3*, as well as the downregulation of *slc2a1*.

We also found benzene exposure induced transcriptomic changes that were closely relevant to fatty acid oxidation, including two shared genes, *pltp* and *mecr*, and 1 ppm exclusive DEGs, *apob*, *serpinf1*, *foxa2*, *c3*, and *hmgcra*. Among the top canonical pathways, LXR/RXR function showed the most statistical significance (−log(*p*-value) = 3.1) and a trend toward activation (Z = 1.0). Zebrafish and mammals have conserved functions in LXR/RXR activation, which regulate the biosynthesis of fatty acid and cholesterol during embryogenesis [58]. In previous studies, benzene exposures increased the expression of fatty acid transport- and β-oxidation-related enzymes in mice [59]. Changes in fatty acid oxidation metabolism were detected in human plasma [60] and mice bone marrow cells following low-level occupational and high-level exposure, respectively. Since fatty acid oxidation is essential for the differentiation [61] and self-renewal [62] of hematopoietic stem cells, as well as the growth and maintenance of leukemic cells [63,64], they may be associated with the molecular mechanisms of benzene hematotoxicity.

Transcriptomic changes of 1 ppm benzene exposure were also related to the perturbation of the immune system during embryogenesis. Previous studies found benzene can dysregulate the cytokine network in vitro and in vivo, potentially through alteration of gene expression, or direct damage of hematopoietic progenitor cells [65,66,67]. In our study, one of the top networks is the crosstalk between PI3k/Akt signaling and PLTP modulation, which is implicated with the alteration of immune cell trafficking and inflammatory activation. The key gene of the oxidative stress response [68], *pik3c2b,* is also involved with PI3k/Akt signaling that regulates immune cell development, differentiation, and function [69]. Several metabolic-related DEGs, including the upregulation of *apob* and downregulation of *pltp*, are implicated with the activation of inflammatory response through PLTP modulation. The *apob* encoding protein is predicted to have low-density lipoprotein particle receptor binding activity and can transfer cholesterol, lipoprotein, and triglycerides. Elevated levels of lipoproteins can trigger the adhesion and migration of immune cells to the endothelial surface and the production of several plasma inflammatory markers, such as the tumor necrosis factor and interleukin [70]. The inflammatory response is strongly affected by circulation and disaggregation of lipoprotein-associated proteins, which can be modulated by PLTP [29]. Another mediator of cytokine homeostasis [45], *foxA2*, was upregulated following benzene exposure in our study. FoxA2 expression can also be upregulated by fatty acids and peroxisome proliferator-activated receptors in skeletal muscle in the mice model [45]. *Nfkbia* is another detoxification gene that is also involved with inflammatory response. It encodes the inhibitor of NF-κB signaling and thus can alter the expression of proinflammatory genes [71]. In our study, *nfkbia* was upregulated following 1 ppm exposure, and we observed that a cytokine gene, *IL-15*, was downregulated simultaneously.

Our study shows that benzene exposure of 1 ppm can induce transcriptomic alterations that are associated with oxidative stress response, fatty acid metabolism, immune system and inflammatory responses, and hematological system development. In addition, the number of dysregulated genes implicated for the top canonical pathways has a monotonic dose–response relationship with benzene levels. Previous studies in humans also show that benzene causes hematotoxic effects following occupational exposure at levels lower than 1 ppm [72,73,74]. Since the disruption of hematological system development is interlinked with benzene metabolism and associated inflammatory responses, our study indicates that zebrafish are great models to evaluate the effects of benzene exposure on transcriptomic changes. Future studies should be conducted to investigate if benzene-induced transcriptomic changes during embryogenesis have long-term consequences at levels equal to or lower than 1 ppm. Our results also demonstrate that the use of experimental zebrafish is reproducible and well-controlled model for VOC exposure studies. Since VOC contaminants often occur in the environment as complex mixtures, the appropriate integration of in vivo, in vitro, and in silico data with epidemiological studies is paramount for robust health risk assessments [75,76].

## Figures and Tables

**Table 1:** Dysregulated genes of benzene exposure based on gene expression fold changes relative to unexposed controls.

Gene Symbol	Gene Name	1 ppm	0.1 ppm
**Inflammatory response**			
*pik3c2b*	Phosphatidylinositol-4-phosphate 3-kinase, catalytic subunit type 2 beta	2.8	1.85
*mep1a.2*	Meprin A, alpha (PABA peptide hydrolase), tandem duplicate 2	1.7	1.73
*chia.2*	Acidic chitinase 2	2.01	1.90
*chrnb2*	Cholinergic receptor, nicotinic, beta 2	0.59	0.54
*pltp*	Phospholipid transfer protein	0.57	0.58
*nfkbiaa*	Nuclear factor of kappa light polypeptide gene enhancer in B-cells inhibitor, alpha a	1.70	
*apob*	Apolipoprotein B	1.70	
*si:ch1073-280e3.1*	Orthologous to human c2, complement c2	1.70	
*c3a.3*	Complement C3a, tandem duplicate 3	2.16	
*il15*	Interleukin 15	0.49	
*ifih1*	Interferon induced with helicase C domain 1	0.54	
*mapk11*	Mitogen-activated protein kinase 11		0.58
**Hematological system development and function**			
*acad11*	Acyl-CoA dehydrogenase family, member 11	3.20	
*antxr2*	ANTXR cell adhesion molecule 2	0.49	
*gabrb1*	Gamma-aminobutyric acid type A receptor subunit beta1	0.58	
*hic1*	HIC ZBTB transcriptional repressor 1	1.72	
*hsp90aa1.1*	Heat shock protein 90, alpha (cytosolic), class A member 1, tandem duplicate 1	1.82	
*kif21b*	Kinesin family member 21B	2.12	
*slc27a2b*	Solute carrier family 27 member 2	1.72	
*slc9a5*	Solute carrier family 9 member A5	0.56	
*lancl2*	Lanthionine synthetase C-like 2	0.59	
**Lipid/fatty acid metabolism**			
*mecr*	Mitochondrial trans-2-enoyl-CoA reductase	2.14	1.97
*cyp2k6*	Cytochrome P450, family 2, subfamily K, polypeptide 6	1.72	
*cyp2n13*	Cytochrome P450, family 2, subfamily N, polypeptide 13	1.84	
*hmgcra*	3-hydroxy-3-methylglutaryl-CoA reductase a	1.94	
*serpinf1*	Serpin family F member 1	1.84	
**Embryogenesis**			
*taf4a*	TAF4A RNA polymerase II, TATA box binding protein (TBP)-associated factor	0.50	0.52
*foxa2*	Forkhead box A2	1.74	
*tacr1a*	Tachykinin receptor 1a	0.58	

## Data Availability

All data is published in this manuscript and Supplementary Materials.

## Supplementary Materials

The following supporting information can be downloaded at: https://www.mdpi.com/article/10.3390/toxics10070351/s1. Table S1: Differentially expressed genes due to benzene exposure. Table S2: Top IPA pathways affected by 1 or 0.1 ppm benzene exposure. Table S3: Estimated Z-score of biological process following 1 ppm benzene exposure.
