# Supplementary material for: Evaluating Phenotypic and Transcriptomic Responses Induced by Low-Level VOCs in Zebrafish: Benzene as an Example
Source: Toxics. 2022 Jun 27;10(7):351. doi: 10.3390/toxics10070351 (PMC9324908; doi:10.3390/toxics10070351)
Supplement: Supplementary file 1 [file toxics-10-00351-s001.zip › toxics-1749112-Table S2 and S3.pdf]

toxics MDPI

Table S2: Top IPA pathways affected by 1 or 0.1 ppm benzene exposure

Benzene exposure levels	1 ppm	0.1 ppm
**Disease and disorders**		
Cancer	134	32
Organismal Injury and Abnormalities	136	32
Gastrointestinal Disease	122	30
Endocrine System Disorders	121	
Reproductive System Disorders	111	
Inflammatory Response		8
Renal and Urological Disease		6
**Molecular and cellular function**		
Cellular Movement	38	13
Small Molecule Biochemistry	43	8
Cell Morphology	15	
Cellular Assembly and Organization	8	
Lipid Metabolism	36	
Cell Cycle		10
Cell-to-Cell Signaling and Interaction		10
Cellular Growth and Proliferation		10
**Physiological system development**		
Hematological System Development and Function	18	
Immune Cell Trafficking	17	
Tissue Morphology	25	
Organismal Survival	43	
Connective Tissue Development and Function	11	
Reproductive System Development and Function		6
Nervous System Development and Function		10
Cardiovascular System Development and Function		7
Embryonic Development		10
Organismal Development		12

Table S3: Estimated Z-score of biological process following 1 ppm benzene exposure

**Biological process**	**Z-score**
**Nervous System Development and Function**	
Cognition	-2.4
Learning	-2.2
Memory	-2.0
**Gastrointestinal Disease**	
Hepatic steatosis	-2.0
**Organismal Development**	
Size of body	2.0
**Infectious Diseases**	
Infection by RNA virus	2.5
**Canonical Pathways**	
Phagosome Formation	1.4
Insulin Secretion Signaling Pathway	1.3
LXR/RXR Activation	1.0
